# Depression, Anxiety, Stress, and Associated Factors among Khat Chewers in Amhara Region, Northwest Ethiopia

**DOI:** 10.1155/2020/7934892

**Published:** 2020-09-26

**Authors:** Seyfe Asrade Atnafie, Niguse Yigzaw Muluneh, Kefyalew Ayalew Getahun, Asegedech Tsegaw Woredekal, Wubayehu Kahaliw

**Affiliations:** ^1^Department of Pharmacology, School of Pharmacy, College of Medicine and Health Sciences, University of Gondar, Gondar, Ethiopia; ^2^Department of Psychiatry, School of Medicine, College of Medicine and Health Sciences, University of Gondar, Gondar, Ethiopia

## Abstract

Individuals with substance use disorder are prone to develop different psychiatric disorders. Substance abuse and associated problems are of current global concern that leads to mental health disorders which contributed about 14% of the global burden of the disease. It has become an epidemic in some parts of the African region with adolescents being the main victims of the ill health and social effects of substance use. This study is aimed at assessing the prevalence of depression, anxiety, and stress and associated factors among khat chewers in the Amhara region, 2019. A community-based cross-sectional study was done from February 14 to April 15, 2019. A purposive sampling technique was used to enroll the subjects. Data was collected using the face-to-face interview technique using the Depression Anxiety Stress Scale 21 (DASS-21) questionnaire. Descriptive statistics and bivariate and multivariate logistic regression were used to summarize the results. *p* value < 0.05 was considered statistically significant. A total of 478 participants were enrolled in the study with a response rate of 94.1%. The overall prevalence of depression, anxiety, and stress was 27.4%, 40.6%, and 18.8%, respectively. Around 43% of the respondents develop dependency from khat chewing. Working in a private sector, being self-employed, being jobless, spending 90 to 180 minutes and more, chewing 51-100 g and more, and chewing khat more than once per week were positively associated with stress. On the other hand, being a private sector worker, being jobless, completing secondary education, earning 1001-5000 ETB per month, chewing khat more than once per week, being khat dependent, and the presence of chronic illness were positively associated with anxiety. History of chronic illness and being khat dependent were positively associated with depression. The prevalence of depression, anxiety, and stress was high among khat chewers in the Amhara region. Special attention has to be given to khat chewers since khat chewing will double the burden of mental illness. Proper awareness and evaluation activities will reduce the impact of the problem.

## 1. Introduction

Mental health disorders according to the World Health Organization (WHO) are one of the leading causes of disability worldwide. Three of the ten leading causes of disability in people between the ages of 15 and 44 are mental disorders [1, 2]. The mental health action plan for 2013–2020, recently published by the WHO, demonstrated the need for collective evidence-based efforts to improve mental health [3]. Severe mental health problems interfere with individuals' emotional, cognitive, and social abilities that can lead to underemployment and reduced productivity. Mental health problems affect society as a whole, and no group is immune to mental disorders [4].

In Africa, mental illness is an important public health challenge that is underrecognized as a public burden. Studies conducted in South Africa revealed that the prevalence of common mental disorders is 27% [5]. In Ethiopia, mental disorders account for 11% of the total burden of diseases [6]. Even if the mental problem was included in the national health policy of Ethiopia, interventions against the problem are very limited and lack of information about the problem is a contributory factor for poor mental health services [7].

Depression, anxiety, and stress levels are considered important indicators for mental health, and the inability to detect and address these psychological disorders negatively affects individuals [8].

Depression disorder presents with depressed mood, loss of interest or pleasure, decreased energy, feelings of guilt or low self-worth, disturbed sleep or appetite, poor concentration, problem of thinking and making decisions, and, in severe stages, recurring thoughts of death or suicide. Among the mental disorders, depression is a disease of global burden affecting 350 million people worldwide [1, 4].

Anxiety is a response of the body to a perceived threat which is triggered by an individual's beliefs, feelings, and thoughts and is characterized by worrying thoughts, tension, increased blood pressure, respiratory rate, and pulse rate, sweating, dizziness, chest pain, and difficulty of swallowing [4]. Anxiety disorders are the most prevalent psychiatric disorders with a current worldwide prevalence of 7.3% [9].

Stress is considered to be a physiological reaction of an organism where diverse defense mechanism comes into play to confront a situation which is perceived as threatening or increased demand [10]. It is also well known that stress is a significant risk factor for the development of drug addiction and addiction relapse [11].

Substance abuse and associated problems are of current global concern that leads to mental health disorders which contributed about 14% of the global burden of the disease. It has become an epidemic in some parts of the African region with adolescents being the main victims of the ill health and social effects of substance use [12]. Substance abuse, including khat chewing, is as old as the history of mankind [13].

Khat (*Catha edulis* Forsk) is a flowering evergreen tree that grows primarily in East Africa and the Arabian Peninsula. Ethiopia is the country of its origin and it is the world's largest khat producer, where khat chewing habit started in the 15th century [14, 15].

Khat contains more than 40 alkaloids, glycosides, tannins, amino acids, vitamins, and minerals. Most of the effects of chewing khat are thought to come from cathinone and cathine—which are structurally related to amphetamine [14, 16]. Cathinone is considered to be the most active ingredient of khat and estimated to be 7–10 times more potent than cathine. Normally, fresh leaves contain a higher proportion of the desirable cathinone that produces sympathomimetic and central nervous system stimulation analogous to the effects of amphetamine [14, 17].

Several million people are chewing khat worldwide, with an estimation of 10 million people chewing khat daily for its euphoric and psychostimulant effect. Studies in different corners of the globe revealed that khat chewing practice had increased prevalence. In Saudi Arabia, the prevalence was between 23.1% and 30.3% [18]. With the recent globalization, khat chewing has spread with African and Arabian immigrants to various Asian and European countries and to Australia as well as to the United States [19, 20]. The users of khat in these new countries are predominantly immigrants from the khat-chewing countries [21].

In Ethiopia, chewing of khat is becoming habitual and increasing at an alarming rate, especially in the younger segment of the population [17]. The overall prevalence of khat use in Ethiopia was 15.3% which was 22.6% among men and 9.1% among women of 15–49 years [22, 23]. The prevalence of khat chewing was variable in different regions of Ethiopia. Khat chewing was highly prevalent in the Harari regional state where more than half of the population (53.2%) chews khat. In the Amhara region, the reported prevalence of khat chewing was 7.8% of the total population and still increasing in prevalence along with emerging cultivation of khat in the region [22].

Khat chewing is one of the most common serious public health concerns affecting the lives of people, particularly the most productive population [23]. Literature shows that the acute effects of khat include increased levels of alertness, enhanced ability to concentrate, friendliness, contentment, and flow of ideas. This is usually followed by excessive tension, anxiety, emotional instability, irritability, and restlessness within 2 hours, followed by feelings of low mood, numbness, lack of concentration, sluggishness, and insomnia [14, 24].

Despite being an established practice, the potential risks and benefits of khat chewing are hotly debated, particularly regarding the association between khat and mental illness. There are many case reports on a possible association between excessive khat use and the occurrence of mental disorders, such as manic-like psychosis and the incidence of psychotic symptoms [25, 26]. Studies pointed out that frequent khat users manifested anxiety, depression, and stress, more repeatedly than nonkhat chewers. Those psychological problems of khat chewing are strongly associated with the severity of dependence scale (SDS) on khat [25, 27]. “The heavier and more frequent the use, the greater the risk was seen to be” [28], but there are also studies that did not show the association between khat use and mental disorders [29, 30]. The aim of this study is to assess depression, anxiety, stress, and associated factors among chronic khat chewers in the Amhara region, Northwest Ethiopia.

## 2. Methods

### 2.1. Study Design

A cross-sectional study was conducted from February 24 to April 15, 2019, in the Amhara region, Northwest Ethiopia.

### 2.2. Setting

The study was conducted in selected cities of the Amhara region. The total population of Ethiopia is 112,640,978 according to the Ethiopian demographic health survey, 2019, of which the Amhara region accounts for 27% of the population. Bahirdar is the capital of the Amhara region and 550 km far from Addis Ababa, the capital city of Ethiopia.

### 2.3. Participants

The source population was all khat chewer residents of the Amhara region whereas the study population was all khat chewers that presented during the study period in the study area. The inclusion criteria are those who chew khat in the last one month. The exclusion criteria are those who were seriously ill.

### 2.4. Variables

The independent variables were age, sex, educational status, religion, marital status, monthly income, dependency, duration of khat chewing, and amount of chewed khat. The dependent variables were depression, anxiety, and stress.

### 2.5. Data Sources

Data were collected by four psychiatry-trained nurses using the pretested interviewer-administered Depression Anxiety Stress Scale 21 (DASS-21) questionnaire [31]. A structured questionnaire was used to collect data through a self-administered questionnaire under close supervision. The interview was also used for those who cannot read and write.

### 2.6. Bias

To assure the quality of data, the data collectors are trained, and the collected data were closely evaluated by the supervisors. The questionnaire was written in English and translated into Amharic by three supervisors, and the research was conducted in Amharic, which is the primary work language of residents of the Gondar town. Finally, the questionnaire was back-translated into English for the final write-up. The questionnaire form was divided into four sections: (a) sociodemographic data (e.g., age, gender, education level, and khat chewing practice), (b) khat chewing habit, (c) SDS-khat, and (d) DASS-21. The questionnaire was distributed to the khat chewers after validating the questionnaire by a pilot study on residents of Woreta town. This validation was done according to geriatric depression scale [32, 33]. The tool Depression Anxiety Stress Scale 21 (DASS-21) was used to measure depression, anxiety, and stress among khat users but not modified or changed. For clarity of vague words of open-ended questions and time taken to complete the questionnaire, a pretest was done on 5% of the total sample before the actual data collection. The sampling method and some questions were changed from the responses of the pilot study.

### 2.7. Study Size

The minimum number of samples required for this study was determined by using a single population proportion formula considering the following assumptions:

(1) $$\begin{array}{c} n_{i}=\frac{\left(Z\alpha /2\right)2p\left(1-p\right)}{d2}, \end{array}$$

where *n* is the minimum sample size required for the study, *Z* is the standard normal distribution (*Z* = 1.96) with a confidence interval of 95% *α* = 0.05, *p* = 28.2% [27], and *d* is the absolute precision or tolerable margin of error (*d*) = 4% = 0.04.

(2) $$\begin{array}{c} n_{i}=\frac{\left(1.96\right)2\times 0.28\left(1-0.28\right)}{\left(0.04\right)2}=484. \end{array}$$

5% of nonrespondents were added to the total sample size to get 508 samples. These 508 samples were proportionally allocated to 7 cities found in 3 zones of the Amhara region. The cities were Bahirdar (166), Merawi (20), Gondar (221), Enfranz (6), Debretabor (59), Addis Zemen (17), and Tisabay (19). The cities were purposely selected for being near the main khat cultivation corridor in the Amhara region, Northwest Ethiopia.

### 2.8. Quantitative Analysis

Descriptive statistics and binary logistic regression were performed to examine the prevalence of mental disorder among khat chewers and the associated factors. Data were cleaned, coded, and entered into SPSS version 20 for analysis.

### 2.9. Statistical Methods

Descriptive analysis was carried out to see the distribution of sociodemographic characteristics, khat use habit, SDS-khat, and DASS-21 score frequencies. Bivariate analysis was performed to find the association of each independent variable with the outcome variables. All variables with a *p* value of 0.20 at bivariate analysis were entered into the multivariate logistic regression model. *p* < 0.05 was considered statistically significant.

### 2.10. Ethical Consideration

During data collection, the appropriate ethical considerations were made. First, ethical clearance was obtained from the Institutional Review Board, University of Gondar, with a reference number of O/V/P/RCS/05/484/2017 to conduct the study. The respondents gave their written consent to participate in the research. Then, respondents were ensured concerning their right to withdraw during participation as well. Since the issue of khat use is relatively sensitive, privacy was maintained during data collection. Those who have depression, anxiety, and stress were informed to get support from the nearby clinic and to consult a psychiatrist or psychologist.

## 3. Result

A total of 508 participants were enrolled in the study with a response rate of 94.1%. The majority of the study participants were male (94.1%) and were between 25 and 30 years of age (46.7%). The childhood residence of most of the respondents (64.7%) was urban area. About 57.5% of the study participants were single, and 35.4% of them completed their secondary education (Table 1).

### 3.1. Khat Chewing Characteristics of Respondents

As shown in Table 2, 38.1% of the respondents chewed khat for 6-10 years. Most of them (60.9%) spent more than 180 minutes for khat chewing per day. About 43% of them chew between 51 and 100 grams of khat and majority of them (57.5%) chew khat daily. In terms of the money spent, around half of the khat chewers (47.9%) spent 25-50 birr per day.

Only one-third of the respondents (33.1%) had a family history of khat chewing, and majority of the family members (57.5%) did not know about the chewing habit of the respondents. The majority of them chew khat with friends (78.0%), and 28.5% of them needed to increase the pattern of khat chewing. Around half of the respondents (46.4%) used khat during work and smoke cigarettes (56%) together with chewing khat. Around two-thirds of the respondents (66.5%) drink alcohol after chewing khat. About 13% of the respondents had a history of chronic illness, and 37% had encountered health problems after they start khat chewing (Table 3).

**Figure 1:**
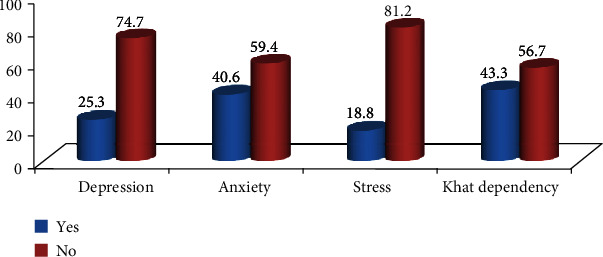
Prevalence of depression, anxiety, stress, and dependency among khat chewers in Amhara region, 2019.

The study reveals that 25.3%, 40.6%, and 18.8% of khat chewers had depression, anxiety, and stress, respectively. The study also reveals that 43.3% of khat chewers had developed khat dependency as shown in Figure 1.

### 3.2. Factors Associated with Khat Chewing and Stress

Binary logistic regression analysis was conducted to identify associated factors to outcome variables like stress, anxiety, and depression. Occupation, number of days of chewing khat per week, and duration of khat chewing were positively associated with stress whereas amount of khat chewed was negatively associated with stress. Khat chewers who work in private sector, are self-employed, and have no job have 3.17 (AOR = 3.17, 95% CI: 1.29, 7.82), 2.48 (AOR = 2.48, 95% CI: 1.08, 5.72), and 2.81 (AOR = 2.81, 95% CI: 1.08, 7.32) times more likely to be stressed as compared to government workers, respectively. The average duration of time for khat chewing was another factor which shows a statistically significant association with stress. Those khat chewers who spent between 90 and 180 min and more than 180 min have 5.91 (AOR = 5.91, 95% CI: 2.27, 15.37) and 2.17 (AOR = 2.17, 95% CI: 1.10, 4.27) times more likely to develop stress as compared to less than 90 min of duration of khat chewing, respectively. Those khat chewers who chew more than once per week have high risk of developing stress as compared with those who chew once per week. However, the amount of khat chewing was found to be a protective factor for stress. Those who chew 51-100 grams and >100 grams of khat were 51% (AOR = 0.49, 95% CI: 0.26, 0.92) and 68% (AOR = 0.32, 95% CI: 0.14, 0.73) less likely to develop stress as compared with <50 grams of khat chewing, respectively (Table 4).

### 3.3. Factors Associated with Khat Chewing and Anxiety

Occupation, education, number of days of chewing khat per week, monthly income, being dependent on khat, and presence of chronic illness were positively associated with anxiety. Those who work in the private sector were 2.51 times (AOR = 2.51, 95% CI: 1.16, 5.41) more likely to be anxious as compared to the government workers, and those who were jobless were 3.78 times (AOR = 3.78, 95% CI: 1.16, 8.55) more likely to be anxious as compared with the government workers. Those who completed secondary education were 0.57 times (AOR = 0.57, 95% CI: 0.33, 0.97) less likely to develop anxiety as compared with those who have college and university education. Those who had a monthly income of 1001-5000 ETB were 3.24 times (AOR = 3.24, 95% CI: 1.03, 10.13) more likely to be anxious as compared with those who earn more than 10000 ETB. Those who chew khat daily had 4.11 times (AOR = 4.11, 95% CI: 1.59, 10.62) more likely to be anxious as compared to those who chew khat once per week. Those who chew khat 4-6 days per week had 3.65 times (AOR = 3.65, 95% CI: 1.28, 10.42) more likely to be anxious as compared with those who chew khat once per week. Those who chew khat 2-3days per week had 3.56 times (AOR = 3.56, 95% CI: 1.32, 9.78) more likely to be anxious as compared with those who chew khat once per week. Those who are dependent on khat were 2.47 times (AOR = 2.47, 95% CI: 1.57, 3.81) more likely to be anxious as compared with those who are nondependent on khat. Those who had a history of chronic illness were 2.42 times (AOR = 2.42, 95% CI: 1.54, 5.32) more likely to be anxious as compared with those who had no history of chronic illness as shown in Table 5.

### 3.4. Factors Associated with Khat Chewing and Depression

Khat chewers with a history of chronic illness were 2.63 (AOR = 2.63, 95% CI: 1.25, 5.56) times more likely to be depressed as compared with those who have no history of chronic illness. Those chewers who were khat dependent using SDS scale were 6.28 (AOR = 6.28, 95% CI: 1.67-23.61) times more likely to be depressed as compared with nondependent khat chewers as shown in Table 6.

## 4. Discussion

Our study is among the first to examine the association between depression, anxiety, stress, and khat use among people in the Amhara region, Northwest Ethiopia. Specifically, we found a higher rate of depression (25.3%) and anxiety (40.6%). Of all khat chewers, 43.3% of them were khat dependent. A study done in Bahirdar showed that the prevalence of khat dependency was 33% which is lower than this study [28]. Similarly, 31% of a group of 204 khat users of Yemeni origin living in the UK fulfilled the DSM-IV criteria for dependence [30]. The difference might be sample size, tool difference, and time variation. SDS score that has been adapted and validated for the study of khat dependence was used. A cutoff point for the SDS scale of 6 was used which is higher than 5 that was used for the Yemeni chewers in the UK [34]. This might be the reason for the lower prevalence of khat dependency in our result as compared to the study done in Yemeni khat chewers living in the UK (51.0) and chewers in Jazan region, Saudi Arabia (52.2%) [35]. However, the interpretation of our SDS results revealed that around half of our chewers (52.2%) were dependent, which is very close to the percent of dependents among Yemeni chewers (51.0%) living in the United Kingdom. The mean SDS score of our chewers was 5.26, which is similar to that of Yemeni chewers living in the United Kingdom (5.5) but higher than that of khat chewers living in the Jazan region, Saudi Arabia (4.3) [34, 35]. This would be explained by the chewing behavior of our chewers, where the majority of them (60.9%) chew for more than 3 h, between 50 and 100 g of khat (43.3%), and on a daily basis (57.5). This confirms the psychological dependence nature of khat chewing habit.

Overall, this study reveals that the prevalence of depression among khat chewers was 25.3%. The prevalence of depression was comparable to the study done in Jimma University staffs, Southwest Ethiopia (22.9%) [27], among medical students in South Africa (26.5%) [36], among mentally ill persons in Southwest Ethiopia (28.5%) [37], among substance users in Jimma town, Southwest Ethiopia (29.0%) [38], and among postsecondary students in Canada for the past 12 months (14.7%) [39]. However, the current study reported lower depression prevalence as compared to that of a study done among university students in Jazan, Saudi Arabia (53.6%) (49), in Nekemte, western Ethiopia (34.7%) [40], and among university students in Egypt (60.8%) [2]. The reasons for the decreased prevalence of depression in the current study might be a difference in study design, study population, sample size, and study tool.

The prevalence of anxiety among khat chewers in the Amhara region was 40.6%. This finding was higher than that of a study done among postsecondary students in Canada within the past 12 months (18.4%) [39], Jimma University staffs, Southwest Ethiopia (19.2%) [27], mentally ill persons in Southwest Ethiopia (8.2%) [37], adolescents and adults in Nekemte town (29.7%) [40], and medical students in South Africa (26.5%) [36]. The reasons for the increased prevalence were due to khat consumption, population difference, and tool difference. However, the current study reported lower anxiety prevalence as compared to a study done among university students in Jazan, Saudi Arabia (65.7%) [41], and among university students in Egypt (65%) [2]. In our study, the majority of the study population (68.4) was not in college or university. This might be the source of variation apart from other sociodemographic characteristics of the study populations.

In our study, the prevalence of stress among khat chewers in Amhara region was 18.8% which is lower than that of a study done among college students in Debrebirhan, central Ethiopia [42], among staffs of Jimma University, Southwest Ethiopia (28.2) [27], among medical students in South Africa (29.5%) [36], among university students in Jazan, Saudi Arabia (34.3) [41], and among university students in Egypt (60.8%) [2]. Like anxiety, university education can increase the level of stress. As a result, the reported prevalence of stress among university or college students was higher.

### 4.1. Factors Associated with Stress

Khat chewers who work in the private sector and who are self-employed and jobless were at higher risk for stress than government workers. The possible explanation might be job-related workload and strict monitoring by the employer which may result in stress. A khat chewer who spent more than one hour is more likely to develop stress as compared with those who spent less than one hour. The possible reason may be spending more time without any work will create a sense of guilty feeling and stress. In our finding, chewing greater than 50 g of khat resulted in less risk of stress as compared with less than 50 g of khat. The possible explanation might be khat chewing may relieve temporal stress. In terms of frequency of khat chewing, those who chew khat more than once a week are more likely to develop stress than those who chew only once a week. The more time spent in chewing khat, the more feeling of guilt and self-blame and this result in frustration and stress in the long run.

### 4.2. Factors Associated with Anxiety

Khat chewers who work in the private sector and those who were jobless were at higher risk for anxiety as compared with government workers. The possible explanation might be the absence of a job, job-related workload, and strict monitoring by the employer which may result in anxiety. In contrast to this, khat chewers who finished secondary education were less likely to develop anxiety as compared to those who finished college and university education.

In terms of monthly income, those with 1001-5000 ETB monthly income were more likely to develop anxiety as compared with those who earn more than 10000 ETB. The possible reason may be wealth and associated lifestyle can be the source of stress.

As the frequency of khat chewing increased, khat chewers were more likely to develop anxiety. The more time spent in chewing khat, the more feeling of guilt and self-blame and this results in frustration and anxiety in the long run. In terms of khat dependency, those who are dependent on khat were more likely to be anxious as compared with nondependent khat chewers. Khat dependents were at higher risk for different psychiatric disorders. Similarly, previous studies show that there is a strong association of khat use, frequency of khat use, or substance use in general with mental distress [7, 27, 37, 39, 40]. Anxiety was strongly associated with dependent khat users in a study done in Bahirdar, Northwest Ethiopia [28].

Khat chewers with a history of chronic illness were more likely to be anxious as compared with those who have no history of chronic illness. The presence of psychiatric disorder in patients with chronic illness will double the burden of the disease; hence, it will create anxiety for the patient.

### 4.3. Factors Associated with Depression

Those who have a history of chronic illness were more likely to be depressed as compared with those who have no history of chronic illness. The presence of depression in patients with a chronic illness will double the burden of the disease; hence, it will create severe depression to the patient. Depression was strongly associated with dependent khat users in a previous study done in Bahirdar, Northwest Ethiopia [28, 40]. The possible reason is that khat stimulates adrenocortical function. Cathinone and cathine, the primary psychoactive ingredients of khat, stimulate the release of cortisol, norepinephrine, and dopamine. Consequently, the respondents experience psychostimulatory effects such as excitement and talkativeness initially. Then, they develop excessive worry, depressed mood, and tension [14]. The other possible justification is the socioeconomic problems due to the increased demand for money to buy khat.

### 4.4. Limitations of the Study

The survey is drawn from a convenience sample, and its results may not be entirely generalizable. The survey questions regarding depression, anxiety, and stress were self-reported, and due to their private and sensitive nature and possible association with social stigma and discrimination, there may be some degree of underreporting by the participants. The cause-effect relationship between khat chewing practice and mental health disorders cannot be established in cross-sectional studies.

## 5. Conclusion

Overall, the prevalence of depression, anxiety, and stress was higher among khat chewers in the Amhara region. Working in a private sector, being self-employed, being jobless, spending more than 90 min, and chewing khat more than once a week were positively associated with stress. On the other hand, being a private sector worker, being jobless, completing secondary education, earning 1001-5000 birr per month, chewing khat more than once a week, being khat dependent, and the presence of chronic illness were positively associated with anxiety. History of chronic illness and being khat dependent were positively associated with depression. Hence, special attention has to be given to individuals who are khat chewers since khat chewing will double the burden of mental illness. Proper awareness creation and evaluation activities will reduce the impact of the problem.

## Figures and Tables

**Table 1:** Sociodemographic characteristics of khat chewer in the Amhara region, 2019 (*N* = 478).

Variables	Frequency	Percentage
Sex		
Male	450	94.1
Female	28	5.9
Age		
18-24	88	18.4
25-30	223	46.7
31-35	82	17.2
36-40	52	10.9
41-45	18	3.8
>45	15	3.1
Religion		
Muslim	107	35.6
Orthodox	284	59.4
Protestant	14	2.9
Others	10	2.1
Childhood residence		
Rural	30	6.3
Small town	125	26.2
Urban	323	67.6
Marital status		
Single	275	57.5
Married	146	30.5
Divorced	57	11.9
Education status		
Unable to read & write	15	3.1
Can read and write	37	7.7
Primary school	106	22.2
Secondary school	169	35.4
College and university	151	31.6
Occupation		
Government	55	11.5
Private sector	112	23.4
Self-employed	178	37.2
Industries	6	1.3
Daily labor	15	3.1
No job	112	23.4
Monthly income in ETB^∗^		
<1000	199	41.6
1000-5000	215	45.0
5001-10000	44	9.2
>10000	20	4.2

^∗^1 USD = 28.62 ETB.

**Table 2:** Khat chewing behaviors among khat chewers in Amhara region, 2019 (*N* = 478).

Variables	Frequency	Percentage
Duration of khat chewing/year		
1-5	170	35.6
6-10	182	38.1
11-15	80	16.7
16-20	34	7.1
>20	12	2.5
Average duration of khat chewing in min/day		
15-90	89	18.6
91-180	98	20.5
>180	291	60.9
Amount of gram in range		
25-50	206	43.1
51-100	207	43.3
>100	65	13.6
Number of days/week		
Daily	275	57.5
4-6 days	68	14.2
2-3 days	109	22.8
Once	26	5.4
Money spent in ETB for khat per day^∗^		
<25	168	35.1
25-50	229	47.9
51-75	36	7.5
76-100	37	7.7
>100	8	1.7
Money spent in ETB for other than khat^∗^		
0-50	353	73.8
51-100	87	18.2
100-150	14	2.9
>150	24	5.0

^∗^1 USD = 28.62 ETB.

**Table 3:** Khat-related behavioral factors among khat chewers in Amhara region, 2019 (*N* = 478).

Variables	Frequency	Percentage
Family history of khat chewing		
Yes	158	33.1
No	320	66.9
Do family members know about your chewing habit?		
Yes	275	57.5
No	203	42.5
Able to buy khat		
Yes	409	85.6
No	69	14.4
With whom do you chew khat?		
Alone	105	22.0
With friends	373	78.0
Chewing khat during work		
Always	257	53.8
Sometimes	85	17.8
Never	136	28.5
Chewing khat increases work performance		
Yes	222	46.4
No	256	53.6
Facing difficulty for buying khat		
Yes	309	64.6
No	169	35.4
Smoking cigarette during chewing		
Yes	268	56.1
No	210	43.9
Drinking alcohol after chewing khat		
Yes	318	66.5
No	160	33.5
History of chronic illness		
Yes	63	13.2
No	415	86.8
Encountered health-related problem after khat chewing		
Yes	178	37.2
No	300	62.8

**Table 4:** Bivariate and multivariate logistic regression of stress and associated factors among khat chewers in Amhara region, 2019.

Variables	Stress		COR (CI = 95%)	AOR (CI = 95%)
	Yes	No		
Age				
18-24	11	77	2.55 (0.69–9.41)	1.19 (0.20-6.89)
25–30	42	181	1.56 (0.47–5.15)	0.95 (0.19-4.68)
31–35	20	62	1.17 (0.34–4.16)	0.72 (0.14-3.69)
6–40	9	43	0.44 (0.26–0.74)	1.64 (0.30–8.74)
41–45	4	14	1.73 (0.45–6.27)	1.48 (0.22–9.96)
>45	4	11	1.00	1.00
Residence				
Rural	9	21	1.00	1.00
Small town	25	100	1.52 (0.61-3.84)	0.73 (0.22-2.43)
Urban	56	268	1.82 (0.77-4.31)	0.98 (0.31-3.07)
Educational status				
Unable to read & write	3	12	0.99 (0.26–3.74)	0.90 (0.18-4.49)
Can read & write	6	31	1.28 (0.49–3.35)	1.23 (0.41-3.69)
Primary	13	93	0.77 (0.87–3.59)	1.55 (0.67-3.57)
Secondary	38	131	0.86 (0.49-1.46)	0.68 (0.35-1.31)
College and university	30	121	1.00	1.00
Occupation				
Government	17	38	1.00	1.00
Private sector	19	93	2.19 (1.03–4.66)∗	3.17 (1.29-7.82)∗
Self-employed	35	143	1.83 (0.93–5.36)	2.48 (1.07-5.72)∗
Industries	0	6	1.32 (0.19-3.42)	1.96 (0.42-6.02)
Day laborer	2	13	2.91 (0.59-14.3)	2.64 (0.42-14.52)
Jobless	17	95	2.47 (1.15–5.35)∗	2.81 (1.08-7.32)∗
Khat dependency status				
Yes	33	174	1.41 (0.88–2.25)	1.70 (0.98-2.95)∗
No	57	214	1.00	1.00
Family history of khat chewing				
Yes	22	136	1.67 (1.02–2.82)	4.10 (1.43–11.77)∗∗
No	68	252	1.00	1.00
Duration of khat chewing in years				
1-5	28	142	1.00	1.00
6-10	35	147	3.62 (1.07-12.23)∗	0.99 (0.51-1.90)
11-15	18	62	3.00 (0.89-10.01)	0.87 (0.38-2.01)
16-20	4	30	2.46 (0.69-8.69)	1.14 (0.29-4.40)
>20	5	7	5.36 (1.15-25.26)∗	0.22 (0.45-1.09)
Average duration of minutes for chewing khat				
15-90	24	65	1.00	1.00
91-180	10	88	4.03 (1.77-9.21)∗∗	5.91 (2.27-15.37)∗∗
>180	56	235	1.92 (1.07-3.45)∗	2.17 (1.10-4.27)∗
Amount of khat in grams				
25-50	25	181	1.00	1.00
51-100	47	160	0.47 (0.28-0.79)∗	0.49 (0.26-0.92)∗
>100	18	47	0.36 (0.18-0.72)	0.32 (0.14-0.73)∗
Number of days spent per week				
Daily	51	224	2.33 (1.01-5.51)∗	4.28 (1.49-12.37)∗
4-6 days	12	56	2.47 (0.89-6.68)	4.78 (1.38-16.54)∗
2-3 days	18	91	2.68 (1.03-6.94)∗	4.69 (1.47-15.00)∗
Once	9	17	1.00	1.00

N.B. ^∗^*p* < 0.05, ^∗∗^*p* < 0.01, and ^∗∗∗^*p* < 0.001; COR = crude odds ratio; AOR = adjusted odds ratio.

**Table 5:** Bivariate and multivariate logistic regression factors associated with anxiety among khat chewers in Amhara region, 2019.

Variables	Anxiety		COR (CI = 95%)	AOR (CI = 95%)
	Yes	No		
Age				
18-24	33	55	0.97 (0.58–1.69)	0.29 (0.61-3.81)
25–30	84	139	0.71 (0.38-1.32)	0.38 (0.88-1.63)
31–35	38	44	0.51 (0.26–1.03)	0.32 (0.72-1.39)
36–40	28	24	1.20 (0.41–3.50)	0.23 (0.52–1.03)
41–45	6	12	1.20 (0.38–3.82)	0.55 (0.99-3.07)
>45	5	10	1.00	1.00
Marital status				
Not married before	107	168	1.64 (0.93-2.91)	1.47 (0.77-2.08)
Married	58	88	1.57 (0.85-2.91)	1.79 (0.90–3.52)
Married before	29	28	1.00	1.00
Educational status				
Unable to read & write	5	10	1.11 (0.36-3.43)	1.38 (0.38-4.43)
Can read & write	13	24	1.03 (0.48–2.18)	0.86 (0.36-2.05)
Primary	45	61	0.75 (0.45–1.26)	0.71 (0.38-1.29)
Secondary	77	92	0.67 (0.23-1.04)	0.57 (0.33-0.97)∗
College and university	54	97	1.00	1.00
Occupation				
Government	28	27	1.00	1.00
Private sector	46	66	1.49 (0.78–2.85)	2.51 (1.16-5.41)∗
Self-employed	77	101	1.36 (0.74–2.49)	1.87 (0.91-3.85)
Industries	3	3	1.04 (0.19-5.59)	1.18 (0.18-7.46)
Day laborer	7	8	1.1 9 (0.38-3.72)	1.68 (0.46-6.15)
Jobless	32	79	2.56 (1.31–5.00)∗	3.78 (1.61-8.55)∗
Monthly income in birr				
<1000	80	119	1.89 (0.71-5.01)	2.38 (0.74-7.700)
1001-5000	84	131	1.94 (0.73-5.10)	3.24 (1.03-10.13)∗
5001-10000	20	24	1.50 (0.49-4.52)	3.45 (0.97-12.21)
>10000	10	10	1.00	1.00
Dependency level				
Yes	146	61	2.48 (1.57-3.38)∗	2.47 (1.57-3.81)∗
No	133	138	1.00	1.00
Family history of khat chewing				
Yes	55	103	1.44 (0.97–2.14)	1.31 (0.84-2.02)
No	139	181	1.00	1.00
Duration of khat chewing in years				
1-5	63	107	1.00	1.00
6-10	75	107	0.84 (0.55-1.29)	0.71 (0.43-1.19)
11-15	37	43	0.68 (0.39-1.17)	0.67 (0.34-1.29)
16-20	14	20	0.84 (0.39-1.78)	0.77 (0.29-2.07)
>20	5	7	0.82 (0.25-2.71)	0.75 (0.17-3.32)
Amount of khat in grams				
25-50	91	115	1.00	1.00
51-100	78	129	1.09 (0.61-1.97)	1.32 (0.82-2.02)
>100	25	40	1.06 (0.64-1.72)	1.21 (0.61-2.41)
Number of days spent per week				
Daily	99	176	4.0 (1.68-9.53)∗	4.11 (1.59-10.62)∗
4-6 days	29	39	3.02 (1.16-7.91)∗	3.65 (1.28-10.42)∗
2-3 days	48	61	2.86 (1.15-7.14)∗	3.56 (1.32-9.78)∗
Once	18	8	1.00	1.00
History of chronic illness				
Yes	17	46	2.01 (1.12-3.63)∗	2.42 (1.54-5.32)∗
No	177	238	1.00	1.00

N.B. ^∗^*p* < 0.05, ^∗∗^*p* < 0.01, and ^∗∗∗^*p* < 0.001; COR = crude odds ratio; AOR = adjusted odds ratio.

**Table 6:** Bivariate and multivariate logistic regression factors associated with depression among chronic khat chewers in the Amhara region, 2019.

Variables	Depression		COR (CI = 95%)	AOR (CI = 95%)
	Yes	No		
Resident				
Rural	12	18	1.00	1.00
Small town	37	88	1.68 (0.73-3.86)	1.76 (0.71-4.39)
Urban	82	241	2.08 (0.95-4.53)	2.36 (0.99-5.61)
Educational status				
Unable to read & write	7	8	0.46 (0.16-1.33)	0.68 (0.21-2.18)
Can read & write	9	28	1.24 (0.54–2.84)	1.30 (0.55-3.10)
Primary	21	85	1.61 (0.89–2.92)	1.64 (0.88-3.04)
Secondary	51	118	0.92 (0.57-1.49)	0.88 (0.53-1.44)
College and university	43	108	1.00	1.00
Substance dependency status				
Yes	41	156	2.71 (1.01–7.23)∗	6.28 (1.67-23.61)∗∗
No	80	191	1.00	1.00
Family history of khat chewing				
Yes	35	123	1.51 (0.97–2.35)	1.55 (0.98-2.43)
No	96	224	1.00	1.00
Duration of khat chewing in years				
1-5	51	119	1.00	1.00
6-10	50	132	1.13 (0.71-1.79)	1.15 (0.71-1.88)
11-15	23	57	1.06 (0.59-1.91)	1.12 (0.60-2.08)
16-20	4	30	3.21 (1.08-9.59)∗	2.94 (0.94-9.19)
>20	3	9	1.28 (0.33-4.950)	1.24 (0.29-5.14)
Amount of khat in grams				
25-50	49	157	1.00	1.00
51-100	66	141	0.67 (0.43-1.03)	0.63 (0.39-0.99)∗
>1000	16	49	0.97 (0.49-1.830)	0.78 (0.39-1.57)
History of chronic illness				
Yes	10	53	2.18 (1.07-4.43)∗	2.63 (1.25-5.56)∗
No	121	294	1.00	1.00
Drinking alcohol				
Yes	88	230	0.96 (0.63-1.47)	0.65 (0.32-1.53)
No	48	117	1.00	1.00

N.B. ^∗^*p* < 0.05, ^∗∗^*p* < 0.01, and ^∗∗∗^*p* < 0.001; COR = crude odds ratio; AOR = adjusted odds ratio.

## Data Availability

The data used to support the findings of this study are included within the article.
